# Oncolytic adenovirus drives specific immune response generated by a poly-epitope pDNA vaccine encoding melanoma neoantigens into the tumor site

**DOI:** 10.1186/s40425-019-0644-7

**Published:** 2019-07-10

**Authors:** Alessandra Lopes, Sara Feola, Sophie Ligot, Manlio Fusciello, Gaëlle Vandermeulen, Véronique Préat, Vincenzo Cerullo

**Affiliations:** 1 Université Catholique de Louvain, Louvain Drug Research Institute, Advanced Drug Delivery and Biomaterials, B-1200 Brussels, Belgium; 2 University of Helsinki, Biocenter 2, Viikinkari 5E, Helsinki, Finland

**Keywords:** Oncolytic adenovirus, Cancer, DNA vaccine, Melanoma neoantigens, Tumor microenvironment

## Abstract

**Background:**

DNA vaccines against cancer held great promises due to the generation of a specific and long-lasting immune response. However, when used as a single therapy, they are not able to drive the generated immune response into the tumor, because of the immunosuppressive microenvironment, thus limiting their use in humans. To enhance DNA vaccine efficacy, we combined a new poly-epitope DNA vaccine encoding melanoma tumor associated antigens and B16F1-specific neoantigens with an oncolytic virus administered intratumorally.

**Methods:**

Genomic analysis were performed to find specific mutations in B16F1 melanoma cells. The antigen gene sequences were designed according to these mutations prior to the insertion in the plasmid vector. Mice were injected with B16F1 tumor cells (*n* = 7–9) and therapeutically vaccinated 2, 9 and 16 days after the tumor injection. The virus was administered intratumorally at day 10, 12 and 14. Immune cell infiltration analysis and cytokine production were performed by flow cytometry, PCR and ELISPOT in the tumor site and in the spleen of animals, 17 days after the tumor injection.

**Results:**

The combination of DNA vaccine and oncolytic virus significantly increased the immune activity into the tumor. In particular, the local intratumoral viral therapy increased the NK infiltration, thus increasing the production of different cytokines, chemokines and enzymes involved in the adaptive immune system recruitment and cytotoxic activity. On the other side, the DNA vaccine generated antigen-specific T cells in the spleen, which migrated into the tumor when recalled by the local viral therapy. The complementarity between these strategies explains the dramatic tumor regression observed only in the combination group compared to all the other control groups.

**Conclusions:**

This study explores the immunological mechanism of the combination between an oncolytic adenovirus and a DNA vaccine against melanoma. It demonstrates that the use of a rational combination therapy involving DNA vaccination could overcome its poor immunogenicity. In this way, it will be possible to exploit the great potential of DNA vaccination, thus allowing a larger use in the clinic.

**Electronic supplementary material:**

The online version of this article (10.1186/s40425-019-0644-7) contains supplementary material, which is available to authorized users.

## Introduction

Tumor immunogenicity is not only patient specific but inherently specific to the individual tumor itself [1]. Cancer vaccines allow the delivery of different tumor associated antigens (TAAs) and neoantigens that can be tailored to the individual tumor [2]. This strategy would overcome the self-tolerance associated to TAAs [1, 3] and the issue of the cancer heterogeneity [4–7]. Indeed, neoantigens represent ideal targets against cancer, due to their specific expression in cancer tissue and the potential lack of side effects, and can be used in the design of cancer vaccines [3]. In particular, cancer DNA vaccines are stable, cost-efficient, easy to manufacture, safe and allow the delivery of different antigens in the same plasmid [8]. The use of CD4 epitopes, in addition to the CD8 epitopes, increases the DNA vaccine activity by activating the T helper (Th) response, as already demonstrated in preclinical essays [9–11]. Indeed, immune recognition of mutation-derived epitopes seems to be mostly driven by CD4 + T cells [12, 13]. However, DNA vaccines alone fail to drive a strong immune response in the tumor, probably due to the highly immunosuppressive tumor microenvironment (TME) [8, 14]. For this reason, DNA cancer immunization usually needs the co-administration of other immunotherapeutic agents able to drive the generated immune response in the tumor border [1, 14]. Among the different combinatorial approaches, the oncolytic viruses (OVs) represent the perfect immunological adjuvant to lead specific T cell response within the TME [15, 16].

OVs are biological agents that selectively infect and kill tumor cells without causing damages in healthy cells [17, 18]. They can generate a strong immune response, including: (i) activation of a systemic pro-inflammatory state, (ii) attraction of cytotoxic immune cell populations to the sites of infection to eliminate virus-containing cells, and (iii) alarming neighboring uninfected cells of viral infection [19]. In particular, Oncolytic Adenovirus (OAd) activates the innate immune system, through the activation of TLRs, NOD2 and other cytoplasmic sensors [20]. However, OAd per se is not able to fully eradicate highly aggressive cancers, but it can be modified to be more immunogenic [20]. We previously studied and validated a modified OAd virus in a murine melanoma cell line, whose genome has been modified to introduce a series of CpG motifs [21]. The TLR9 activation that resulted from the presence of CpG inside the virus allowed the activation of NK cells and cytokine production that were responsible of the tumor regression in nude mice [21].

We hypothesized that the combination in a no heterologous prime-boost manner of a poly-epitope and tumor-specific pDNA vaccine with a modified CpG-rich OAd could generate a full and specific immune response both systemically and in the TME. In this purpose, we designed new DNA vaccines encoding different B16F1-TAAs and B16F1-specific neoantigens inserted in a VSV-G gene sequence. VSV-G is a viral protein well known to improve the immune response [22]. The efficacy of a pVAX2 DNA vaccine encoding one CD4 and one CD8 epitope in the VSV-G sequence has been validated in different tumor models, including B16F10 melanoma [23]. Furthermore, the plasmid containing a single epitope demonstrated a selective activation of the CD4 response, when a CD4 epitope is encoded, or the CD8 response, when a CD8 epitope is encoded (Vandermeulen et al., in preparation). The advantage of delivering neoantigens would be to overcome the immune tolerance associated to the TAAs.

In this study, we aimed to improve the DNA vaccine efficacy by combining the poly-epitope DNA vaccines (here called pDNA) and an oncolytic adenovirus serotype 5-CpG (here called OAd) to drive in the TME the antigen-specific immune response generated by the DNA vaccine. To our knowledge, this is the first time that a DNA vaccine and an OAd are combined in a no heterologous prime-boost manner to induce the eradication of an already established tumor. We also explored the mechanisms and the contribution of each therapy to the observed tumor regression, which, until now, has been poorly explored, especially in the context of oncolytic virus treatment.

## Results

### CpG-enriched oncolytic adenovirus and poly-epitope pDNA vaccine synergy enhanced tumor regression in melanoma-bearing mice

**Fig. 1:**
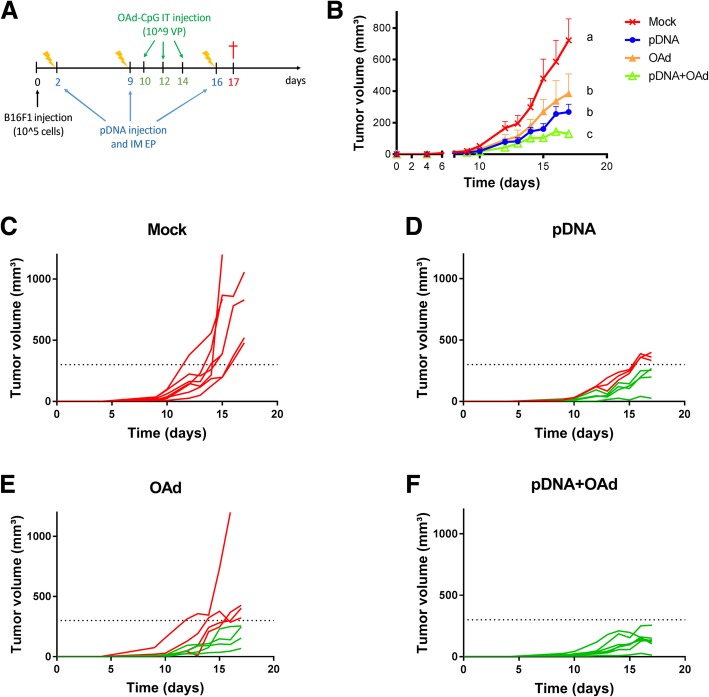
In vivo *pDNA and OAd combination*. **a** Therapeutic DNA vaccination protocol in combination with oncolytic virus therapy. B16F1 cells were injected at day 0; pDNA vaccine was intramuscularly (IM) injected and electroporated 2, 9 and 16 days after tumor injection, while 10^9^ OAd virus particles (VP) were IT injected 10, 12 and 14 days after tumor injection. Mice were sacrificed at day 17. **b** Evolution of tumor volume (mm^3^) after B16F1 challenge as a function of time (days) (mean ± SEM). All the groups were statistically compared to the others using two-way ANOVA, column factor (*p* < 0.05, *n* = 8). **c**-**f** Tumor growth measurement for the single mouse and for each group of mice. In red, mice that developed a tumor >300mm^3^ in volume; in green, mice with a tumor volume < 300 mm^3^. The a, b and c letters indicate statistical differences: the presence of two different letters in two groups indicate a statistical difference (p < 0.05) between them; the same letter in two different groups indicates the absence of a statistical difference between these two groups

To improve the efficacy of DNA vaccination, a pDNA vaccine made of a mix of 4 plasmids encoding 2 melanoma TAAs and 3 neoantigens (Additional file 1) was injected and electroporated in the tibialis muscle of mice and combined with a CpG-enriched OAd virus [21], administered intratumorally (IT) (Fig. 1a). Neoantigen mutations have been detected in the genomic DNA (gDNA) of the B16F1 cells at different passages to verify the stability of the mutations for the therapeutic vaccination. These neoantigens were specifically redesigned according to the mutations found in B16F1 cell line (Table 1).

**Table 1:** antigen nucleotide and peptide sequence and MHC specificity (reactive T cell subtype [12])

Antigen name	Reactive T cell subtype [12]	Nucleotide sequence in B16F1 cells	Peptide sequence
TRP2	CD8	AGCGTGTACGACTTCTTCGTGTGGCTG	SVYDFFVWL
Pbk	CD8	GACAGCGGCAGCCCTTTTCCTGCTGCCGTGATCCTGAGAGATGCCCTGCACATGGCTAGAGGCCTGAAGTACCTGCACCAA	DSGSPFPAAVILR**D**ALHMARGLKYLHQ
Kif18b	CD4	CCTAGCAAGCCCAGCTTCCAAGAGTTCGTGGACTGGGAGAACGTGTCCCCTGAGCTGAACTCTACCGACCAGCCTTTCCTG	PSKPSFQEFVDWE**N**VSPELNSTDQPFL
Cpsf3l	CD4	GAGTTCAAGCACATCAAGGCCTTCGACAGAACCTTCGCTGACAACCCCGGACCTATGGTTCGACCTTGGCAGTCTGCTAGC	EFKHIKAFDRTFADNPGPMV**RPWQSAS** *Sequence in B16F10 cells* [12]*:* EFKHIKAFDRTFA***N***NPGPMV***VFATPGM***
Gp100	CD4	TGGAACAGACAGCTGTACCCCGAGTGGACCGAGGCCCAGAGACTGGAT	WNRQLYPEWTEAQRL

List of the antigens used in the study. Mutated amino acids of the peptide sequence compared to the wild type sequence are in bold and underlined in the table. Mutated amino acids of the gene Cpsf3l in the B16F10 cells compared to the wild type sequence are in bold and not underlined (the sequence in the table is the sequence found in B16F10 cells by Kreiter et al. [12])

Compared to the neoantigens described in the literature for the B16F10 cell line [12], the Cpsf3l sequence that we found in the B16F1 cells showed some differences, as described in Table 1.

To evaluate the therapeutic efficacy of the combination, the tumor growth was followed for each group (Fig. 1b) and for every single mouse (Fig. 1 c-f). All the mice developed a tumor 9 days after the B16F1 cell injection. In the pDNA+OAd group, tumor growth was significantly slower compared with the control groups mock, pDNA or OAd (Fig. 1b). To determine if this effect was additive or synergic, we used the Spector formula [24]. In particular, we calculated the reduction in the tumor volume at day 15 (when the tumor growth curves are significantly different) and we calculated the total standard error (SE) and the combination index (CI), as described in Spector’s study [24]. As our CI was >2SE, we concluded that the effect was synergic (see materials and methods). When the vaccine was combined with the viral therapy, the tumors never reached 300 mm^3^ in volume until the end of the experiment (day 17), as shown in Fig. 1f, while in all the other groups, 46–100% of mice developed a bigger tumor (red lines in Fig. 1 c-e).

### pDNA and OAd increased the immune cell infiltration in the TME; their combination allowed higher antigen-specific T cell infiltration and NK activity

**Fig. 2:**
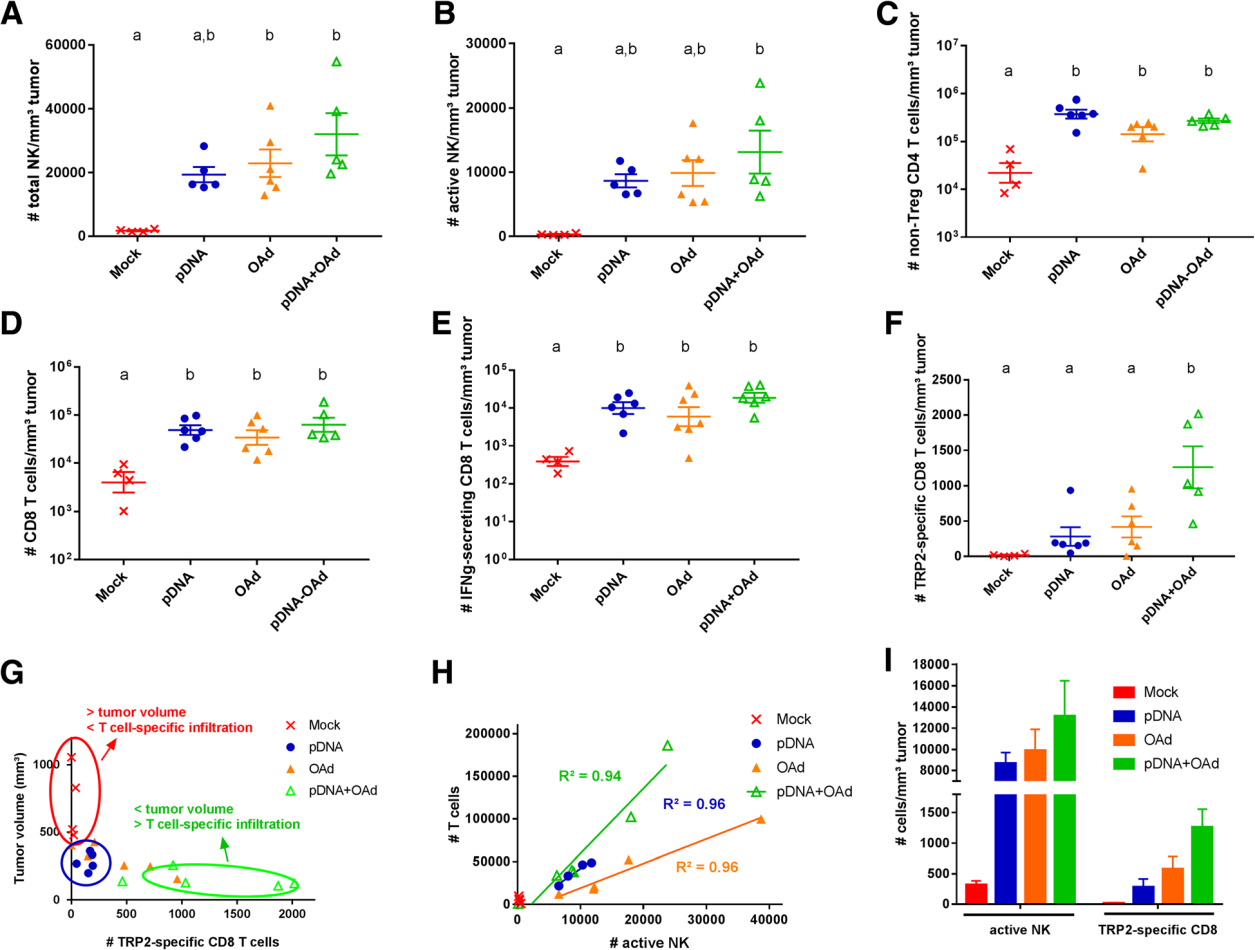
Immune cell infiltration in the TME. **a** Number of total NK cells/mm^3^ tumor. **b** Number of CD335+ (active) NK cells/mm^3^ tumor. **c** Number of non-Treg CD4 T cells/mm^3^ tumor. **d** Number of CD8 T cells/mm^3^ tumor. **e** Number of IFNg-secreting CD8 T cells/mm^3^ tumor. **f** Number of TRP2 antigen-specific CD8 T cells/mm^3^ tumor. **g** Correlation between the tumor volume and the number of TRP2 antigen-specific CD8 T cells in the TME. **h** Correlation between the number of active NK and CD8 T cells. R^2^ was calculated by using a linear regression analysis. **i** Correlation between active NK and TRP2-specific CD8 T cells. The results in **a**, **b**, **c**, **d**, **e**, **f** and **i** are expressed as mean ± SEM (*n* = 4–6). a and b letters on the graphs indicate significantly different results when the superscript letters are different. The annotation “a,b” indicates no significant differences compared to a and b statistical groups. The presence of two different letters in two groups indicate a statistical difference (p < 0.05) between them; the same letter in two different groups indicates the absence of a statistical difference between these two groups

To study the mechanism underpinning the synergy between the plasmid and the oncolytic virus therapy (pDNA+OAd group), infiltration of immune cells was assessed in the tumors of mice 17 days after the tumor injection (Fig. 2). We first assessed the NK cells at the tumor, given their important role in innate rejection of tumor. [25] Interestingly, we found that the total amount of NK cells was increased in all the treated groups and, significantly, in the groups treated with the virus (OAd and pDNA+OAd, Fig. 2a). The number of NK cells that express the CD335 activating receptor [26] (active NK) was significantly higher only in the combination group compared to the mock (Fig. 2b). This data is particularly interesting as it has been so far very poorly studied in the context of oncolytic virus treatment. The number of CD4 and CD8 adaptive immune cells also increased in the treated groups (Fig. 2c and d), as well as the number of IFNg-secreting CD8 T cells (Fig. 2e). As the pDNA vaccine encoded the TRP2 melanoma antigen, the number of TRP2-specific CD8 T cells was evaluated to test the ability of the vaccine to produce an antigen-specific immune response. As TRP2 is encoded in 3 of the four plasmids, it was chosen as the representative epitope to test the antigen-specific T cell infiltration. When mice were treated with the single therapies (pDNA or OAd), the number of TRP2-specific T cells was not increased compared to the mock group. Only when the two therapies were combined, the amount of TRP2-specific T cells was found to be significantly higher compared to all the other groups (Fig. 2f). Different correlation analyses were performed to find the contribution of the different immune populations in the tumor growth. Almost no TRP2-specific T cells were found in bigger tumors (mock group), while a higher infiltration was found in smaller tumors (Fig. 2g). Furthermore, a linear correlation (R^2^ > 0.90) was observed between the number of CD8 T cells and active NK for all the treated groups (Fig. 2h), but also between the TRP2-specific T cells and active NK cells for the combination group (Fig. 2i).

### Cytokine involved in NK and CD8 recruitment and activity are highly expressed in the TME of pDNA+OAd-treated mice

**Fig. 3:**
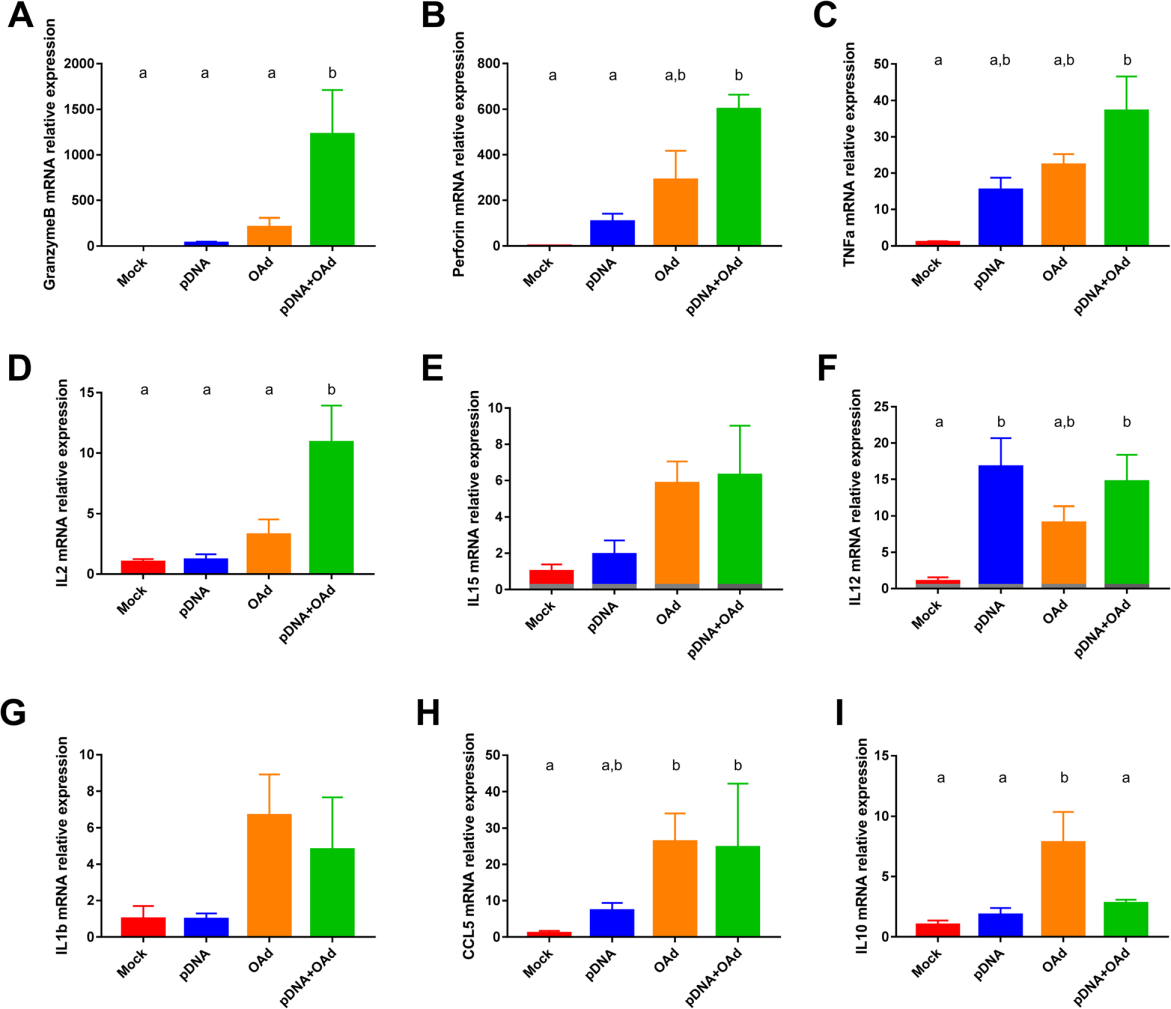
Cytokine expression in the TME. Granzyme B (**a**), Perforin (**b**), TNFa (**c**), IL2 (**d**), IL15 (**e**), IL12 (**f**), IL1b (**g**), CCL5 (**h**), IL10 (**i**). All the results are expressed as mean ± SEM (n = 4–6). a and b letters on the graphs indicate significantly different results when the superscript letters are different. The presence of two different letters in two groups indicate a statistical difference (p < 0.05) between them; the same letter in two different groups indicates the absence of a statistical difference between these two groups. The annotation “a,b” indicates no significant differences compared to a and b statistical groups

To evaluate the activity and the contribution of the NK and T cells in the TME, an evaluation of the cytokine and perforin/granzymeB expression was performed. A general increase in the cytokine expression was observed when mice were treated with both pDNA and OAd (Fig. 3). In particular, a higher expression of proteins related to NK, Th1 and CTL activity was observed, such as Granzyme B [27] (Fig. 3a), perforin [28] (Fig. 3b), TNFa [29] (Fig. 3C) and IL2 [30] [31] (Fig. 3d). Many other interleukins were overexpressed, such as IL15, IL12 and IL1b (Fig. 3e-g). Finally, an increased CCL5 expression was observed in the OAd-treated groups, OAd and pDNA+OAd (Fig. 3h). Generally, all the cytokines, chemokines and enzymes were also overexpressed in the OAd group, but without observing significant differences compared to the mock group. Interestingly, IL10 was significantly overexpressed only in the OAd group (Fig. 3i). This was the only cytokine expressed in higher amount in OAd group but not in the pDNA+OAd group.

### pDNA and OAd induced higher immune cell infiltration in the spleen and the combination induced a greater antigen-specific immune response

**Fig. 4:**
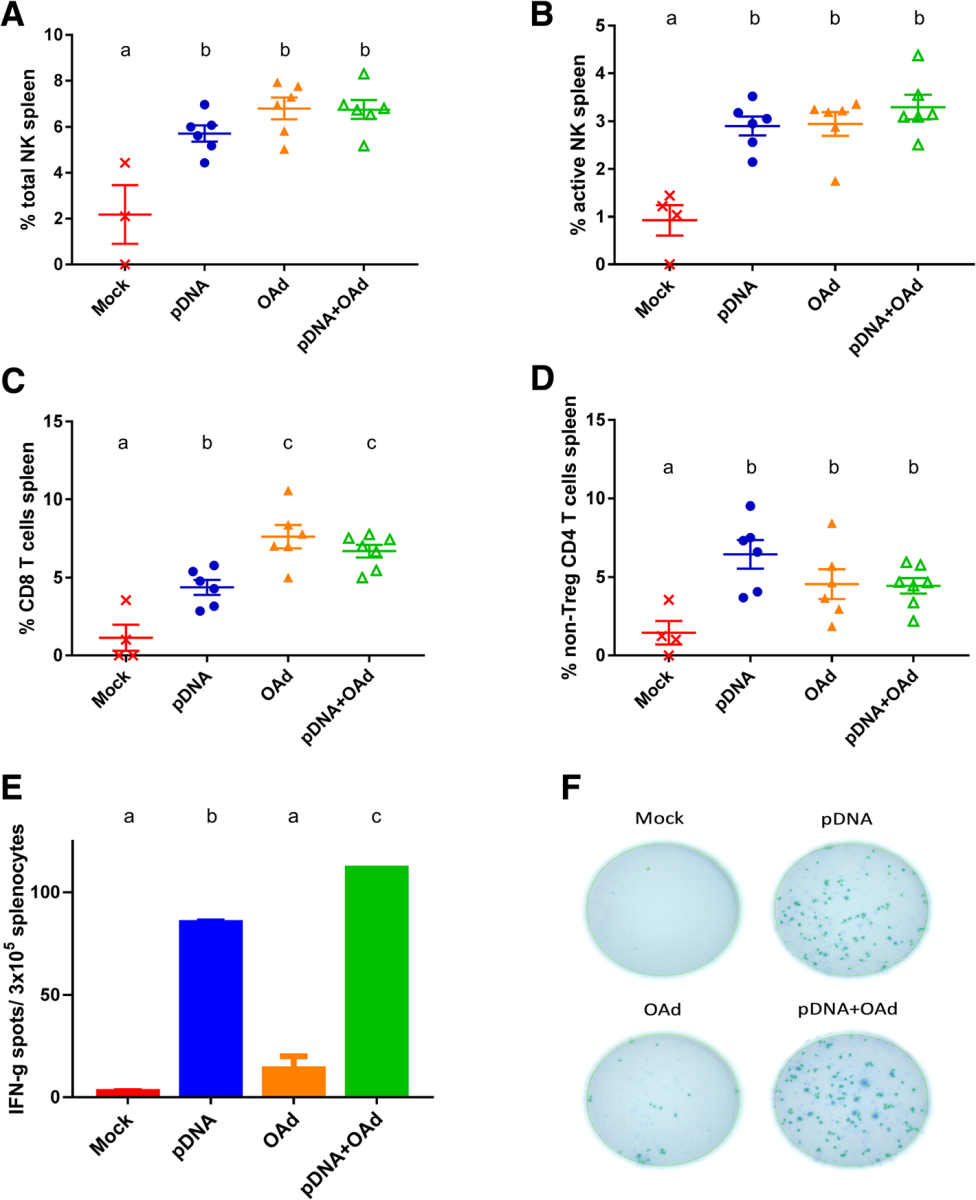
Immune cell analysis in the spleen. **a** Percentage of total NK. **b** Percentage of active NK. **c** Percentage of CD8 T cells. **d** Percentage of non-Treg CD4 T cells. **e**-**f** ELISPOT analysis of the splenocytes stimulated with TRP2 peptide. All the results are expressed as mean ± SEM (n = 4–6). **a**, **b** and **c** letters on the graphs indicate significantly different results when the superscript letters are different. The presence of two different letters in two groups indicate a statistical difference (p < 0.05) between them; the same letter in two different groups indicates the absence of a statistical difference between these two groups

Next, we wanted to evaluate the systemic immune activity and compare it with the response in the TME. To this end, splenocytes were collected 17 days after the tumor challenge and analyzed (Fig. 4). All the treatments significantly increased not only NK infiltration but also the number of active NK in the spleen, compared to the mock group (Fig. 4a and b). In addition to that, the number of CD8 and non-Treg CD4 T cells was higher in the treated groups (Fig. 4c and d). In particular, OAd and pDNA+OAd group showed the highest number of CD8 T cells (Fig. 4c). ELISPOT analysis revealed high TRP2 specificity for pDNA and pDNA+OAd conditions, which was significantly different compared to the other groups (Fig. 4e and f). pDNA+OAd group showed a significantly higher number of IFNg-secreting and TRP2-specific splenocytes compared to pDNA alone (Fig. 4e and f).

### The combination of pDNA and OAd increased the long-term survival

**Fig. 5:**
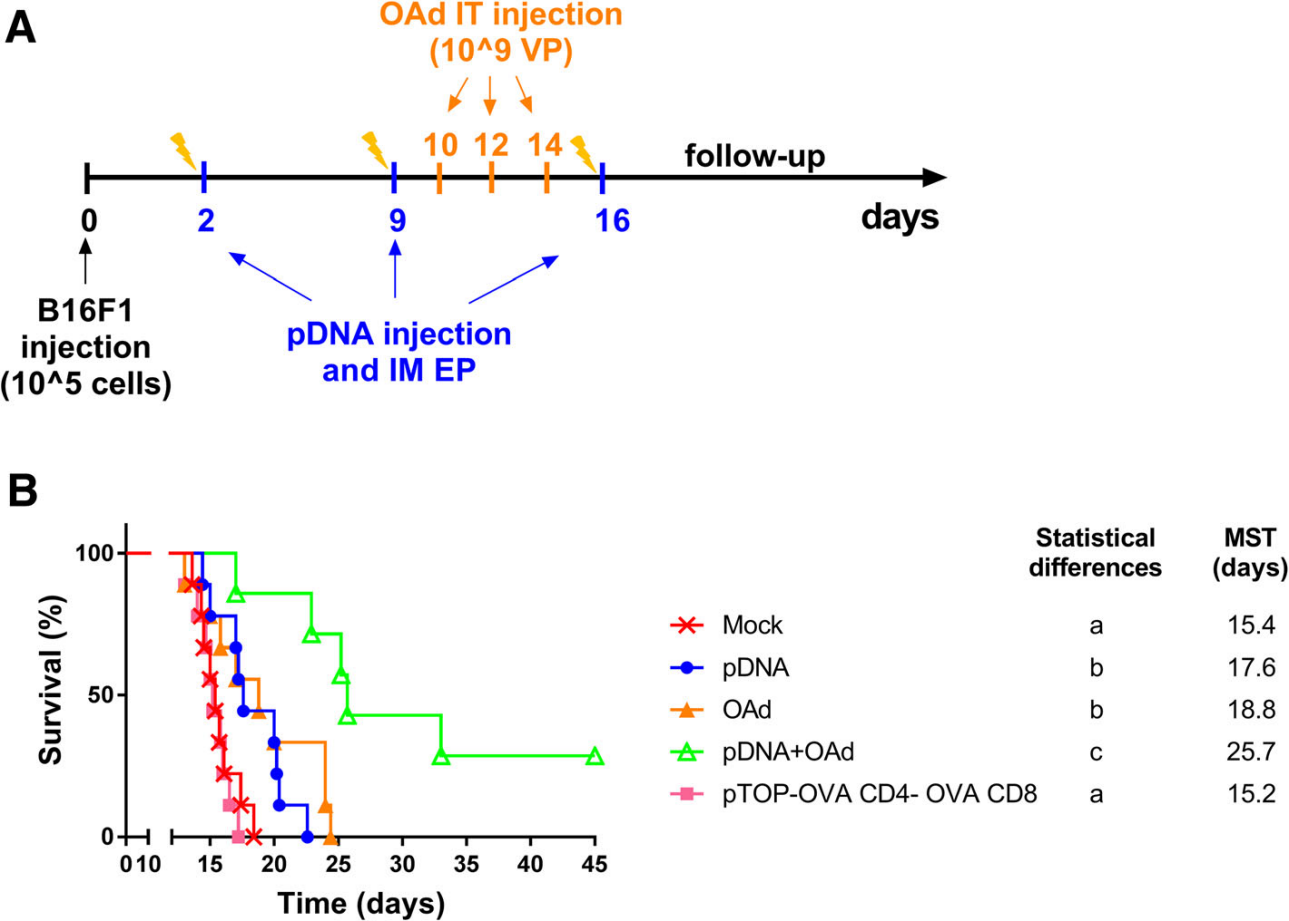
Evaluation of the long-term survival. **a** Therapeutic DNA vaccination protocol in combination with oncolytic virus therapy. B16F1 cells were injected at day 0; pDNA vaccine was intramuscularly (IM) injected and electroporated 2, 9 and 16 days after tumor injection, while 10^9^ OAd virus particles (VP) were IT injected 10, 12 and 14 days after tumor injection. **b** Survival curves representing the percentage of alive mice (%) as a function of time (days); MST = median survival time. Statistical analysis: Log-Rank (Mantel–Cox) test (*p* value < 0.05; *n* = 7–9). The presence of two different letters in two groups indicate a statistical difference (p < 0.05) between them; the same letter in two different groups indicates the absence of a statistical difference between these two groups

Previously, we have addressed the short-term efficacy of the combination between pDNA and OAd, showing a significant decrease in the tumor growth and a higher CTL infiltration and activity when the two therapies are combined. To better understand the o, we performed a new experiment to follow-up the long-term survival. The vaccine and the virus have been administered following the same protocol used to evaluate the tumor growth, but this time, mice were followed-up until day 45 (Fig. 5a). Our results showed that the median survival time (MST) was significantly longer in the group treated with pDNA and OAd. Furthermore, this combination cured almost 30% of mice (2/7 mice), compared to 0% in the other groups (Fig. 5b).

To exclude an unspecific systemic effect of our vaccine, we evaluated the efficacy of the pDNA compared to an irrelevant plasmid. To this aim, a group of mice has been treated with a plasmid encoding for chicken ovalbumin-derived epitopes restricted for CD4 and CD8 in the VSV-G sequence (pTOP-OVA CD4-OVA CD8). This group did not show any differences compared to the untreated mock group, thus supporting the contribution of the adaptive immune effect induced by the pDNA encoding B16F1 TAAs and neoantigens.

## Discussion

In the field of cancer immunotherapy, DNA vaccines showed many promises but also failures due to their poor immunogenicity, especially in the clinic [32]. A renovated interest was aroused by the use of “personalized” and poly-epitope DNA vaccines encoding different TAAs and neoantigens and the possibility to combine them with other strategies that can drive the generated immune response in the TME [14, 33]. In the current study, a DNA vaccine (pDNA) against B16F1 melanoma has been tested. This pDNA was a mix of four different plasmids globally encoding three melanoma neoantigens, specifically designed in silico according to the B16F1 mutations (Kif18b, Cpsf3l and Pbk), and two TAAs (TRP2 and gp100). The neoantigens Kif18b and Pbk presented the same mutations described in the literature for B16F10 melanoma [12, 34], while the Cpsf3l neoantigen sequence that we found in B16F1 cells was different (Table 1). Seven amino acids were found to be mutated in the Cpsf3l gene when compared to the wild type sequence. These results confirm the high mutational burden of melanoma that many authors describe [12, 34–37]. These neoantigens were selected based on the results obtained by other researchers. In particular, the mutated form of Kif18b (K739 N) was found to be a dominant mutated antigen, and mice immunized with mutated Kif18b peptide could slow tumor growth and improve survival [34]. Also a mutated form of Cpsf3l (D314N) has been shown to induce a strong immune reaction preferentially against the mutated peptide [34]. Furthermore, the three selected neoantigens, including the mutated Pbk (V145D), induced an immune response after vaccination with RNA. [38] In particular, Pbk has been described as a CD8 neoantigen, with a low MHC I score, which predicts the binding affinity to the MHC I complex [38].

The antitumor activity of the pDNA vaccine was drastically improved by combining it with an OAd virus whose genome was enriched with CpG motifs (Fig. 1) [21].

We hypothesized that the OAd virus injected IT could activate the innate immune response locally and recruit in the TME the adaptive immune cells generated by the pDNA vaccine. Indeed, the IT viral therapy enhanced the recruitment of NK cells in the TME (Fig. 2a) through the local production of several cytokines and chemokines, such as CCL5, IL15, IL1b, IL10, TNFa, among others (Fig. 3) [29, 39, 40]. It has been already demonstrated that the CpG-enriched OAd could stimulate the TLR9 response and that the anti-tumor immune response was related to the NK recruitment and activity [21]. In particular, beside the higher number of NK cells in the TME, a significantly increased infiltration of CD335+ NK cells (active NK cells) was observed only in the combination group compared to the mock (Fig. 2b). CD335 was originally identified as a receptor with the ability to mediate the killing of tumor-transformed cells. This receptor is also involved in the control and elimination of several pathogens and has a role in immune homeostasis by regulating the expression of several immune cell types [41]. The increased tumor infiltration of CD335+ NK cells can be correlated with a higher granzymeB, perforin and TNFa secretion in the combination group (Fig. 3a-c), as their cytotoxic activity is mainly associated to CTL and NK cells [40, 42]. Specifically, granzymeB plays a critical role in triggering apoptotic cell death, while perforin plays an important role in NK cell-mediated suppression of tumor initiation and metastasis [42]. The significant increase in the number of active NK observed only in the combination group indicates the involvement of the pDNA in this response. Indeed, pDNA alone significantly increased IL12 expression compared to the mock (Fig. 3f). Furthermore, contrarily to the OAd group, pDNA did not increase the IL10 levels (Fig. 3i). These two ILs are important for the NK recruitment/activity. IL12 activates NK cells to destroy a variety of tumors in a perforin-dependent manner [40]. IL10 plays a double role in the TME: from one side, it recruits NK cells, hence initiating immune cell infiltration. However, an excessive IL10 production can prevent the NK activity and transform the Th response in a Th2 response [43]. The low levels of IL10 and the increased expression of IL12 in the pDNA-treated group confirm the ability of the DNA vaccines to shift the immune response towards a Th1 phenotype [29, 44, 45]. IL12, as well as TNFa, not only activate NK cells but are also secreted in response to the innate immune activation to recruit the cells of the adaptive immunity [29, 45]. The high IL12 secretion following pDNA vaccination could be related to the Th1 switch induced by the vaccine itself, probably strengthen by the presence of a CD4 epitope in the pDNA.

Another important cytokine in the TME was IL2. This last is produced predominately by antigen-simulated CD4 T cells, CD8 cells, NK and activated DCs [30]. It plays a critical role in the differentiation of CD4 T cells into a variety of subsets, recruits Tregs, and promotes CD8 T cell and NK cytotoxicity activity [46]. In preclinical and clinical studies, IL2 is administered in combination with cancer vaccines to dramatically enhance their anti-tumor activity [46, 47]. It has been demonstrated that the Tregs recruitment induced by IL2 could be reduced by co-administering IL12 [48]. This association further stimulates Th1, CTL and NK in a positive loop [47]. In the current study, the pDNA vaccine itself permitted to increase the levels of IL12, which in turn could have enhanced the antitumor properties of IL2, contributing to the global antitumor efficacy. Indeed, the involvement of NK, CD8 and CD4 T cell influences the cancer immunity cycle in several aspects. In particular, vaccine-induced CD4 T cells promote an inflammatory tumor microenvironment, by producing IFNg, which improves CTL killing activity and sensitizes tumor cells for recognition and direct killing by cytotoxic Th1 effectors [7]. This cycle will broad the antitumor T cell repertoire and restore the cancer immunity cyle [7].

Finally, the higher NK activity in the TME was directly correlated with the number of TRP-2 specific CD8 infiltrated in the TME (Fig. 2i), which was inversely correlated with the tumor volume (Fig. 2g). All these effects can explain why the NK cells are significantly more active in the TME and the immune response is stronger only when the two therapies are combined.

In the spleen, both pDNA and OAd had an effect in increasing NK, CD4 and CD8 cells (Fig. 4a, b). In particular, antigen-stimulated splenocytes produced significantly higher amount of IFNg in the combination group, compared to all the other groups (Fig. 4e, f). pDNA mainly contributed to this effect by generating a high amount of TRP2-specific T cells. This means that the vaccine can induce the production of antigen-specific T cells systemically, but it cannot drive the mounted immune response in the TME, probably due to the immunosuppressive TME. The concomitant IT administration of the virus can activate the innate immunity and recall the antigen-specific T cells generated by the vaccine into the tumor site. For this reason, we found an increased infiltration of TRP2-specific CD8 cells only in the TME of the combination group. This result confirms our previous study in a mastocytoma tumor model, where we observed that the DNA vaccine alone failed to significantly increase the survival, due to a poor immune T cell infiltration in the TME. Only when it was combined with immune checkpoint blockades the antitumor efficacy was significantly enhanced, due to the higher specific T cell infiltration and activity induced by the immunocheckpoint blockers [14].

Globally, these results explain the drastic decrease of the tumor growth rate and the significant improved survival observed when pDNA was combined with the OAd viral therapy, thus curing 30% of mice from a lethal melanoma cell injection.

## Conclusions

In this study, we demonstrated that the potency of a poly-epitope cancer DNA vaccine against melanoma could be dramatically enhanced by the combination with an oncolytic virus administered in the tumor. The reason of the improved efficacy was the complementarity of the two therapies in activating the immune system. From one side, the local IT viral therapy was able to recruit NK innate immune cells in the TME. Therefore, different cytokines, chemokines and enzymes involved in the adaptive immune system recruitment and in the cytotoxic activity against the tumor were produced. On the other side, pDNA was able to produce antigen-specific T cells in the spleen, which reached the tumor when recalled by the local viral therapy. pDNA played also an important role in IL12 production in the TME, which created a positive loop in cytokine production and immune cell activity [47]. The global effect was the dramatic slowdown in the tumor growth and a significant increase in the survival observed in the combination group compared to all the others. Our study demonstrates that a rational combination therapy involving DNA vaccination could overcome its poor immunogenicity in the TME, leading the way to a wider use of DNA vaccination in humans.

## Materials and methods

### Cell lines

B16F1 cells, a melanoma cell line from C57BL/6 mice, were purchased from American Type Culture Collection (ATCC; Manassas, Virginia) and cultured in MEM complete medium, containing 10%FBS, 100 μg/ml streptomycin and 100 U/ml penicillin (Life Technologies, California). The human lung carcinoma cell line A549 were purchased from ATCC and cultivate in DMEM supplemented with 10% FBS, 1% glutamine, 100 μg/ml streptomycin and 100 U/ml penicillin.

### gDNA extraction and detection of B16F1 neoantigen mutations

B16F1 neoantigens were selected from immunogenic B16F10 mutations described in the literature, according to their low MHC I score, which indicates a high binding affinity, the response to RNA vaccination and the reactive T cell subtype (CD4 or CD8) [12]. The presence of three selected neoantigens was verified in B16F1 cell line. Specific mutations were detected in the gDNA of the cells at passage number 7, 16, and 27 to verify the presence of the mutations at different time points (passage number 1 is when cells were purchased from ATCC). gDNA was extracted using the PureLink™ Genomic DNA Mini Kit (Thermo Fisher, Massachusetts). Briefly, 20 μl of Proteinase K and 20 μl of RNAseA were added to 200 μl of cells (10^6^ cells) and incubated for 2 min at room temperature (RT). Then, 200 μl of the Lysis/binding buffer were added to the cells and incubate at 55 °C for 10 min, before adding 200 μl of pure ethanol. The lysate was purified using a spin column after 2 steps of washings with the washing buffers provided in the kit. gDNA was collected using 50 μl of elution buffer and stocked at − 20 °C, before performing the PCR amplification (see section 5.8). Sanger sequencing was performed by Genewiz (United Kingdom) to verify the presence of specific mutations.

### pDNA vaccine design and production

Four different plasmids encoding melanoma antigens TRP2, Pbk, Kif18b, Cpsf3l and human gp100 were designed (Additional file 1). Among the chosen antigens, 3 are recognized by MHC class II (Gp100, Kif18b and Cpsf3l), to stimulate CD4 T cell response, while the others belong to MHC class I epitopes (TRP2 and Pbk). The antigens TRP2 and gp100 are already known melanoma antigens [49, 50]. Their presence has been verified in B16F1 cell line (Additional file 2). The other three are neoantigens described in the literature for the B16F10 melanoma cell line [12]. They have been chosen among the B16F10 immunogenic mutations, based on their MHC class I binding (low score for higher affinity) and their response after RNA and peptide vaccination in B16F10 tumor model [12]. These neoantigens were specifically redesigned according to the mutations found in B16F1 cell line (Table 1). The mutations were verified at different B16F1 cell passages and compared with the gene expression in the spleen of C57Bl/6 mice as a non-mutated control. They have been cloned in a pVAX2 vector encoding the VSV-G viral protein. Two antigens for each plasmid, one CD4 and one CD8, were inserted inside the VSV-G sequence, as described in [23]. Four plasmids were obtained: pTRP2-Gp100; pTRP2-Cpsf3l; pTRP2-Kif18b; pGp100-Pbk (Additional file 1). The mix of the four plasmids in a 1:1:1:1 proportion (1 μg for each plasmid) was called pDNA. In Table 1, the nucleotide and peptide sequences of each antigen are shown. The underlined amino acids indicate the presence of a mutation specific in B16F1 melanoma cell line.

As an additional control for the survival experiment, an irrelevant plasmid has been used, encoding two OVA epitopes in the VSV-G sequence (one CD4 and one CD8 epitopes). This plasmid has been called “pTOP-OVA CD4-OVA CD8”.

### Virus production

Ad5D24-CpG is an OAd bearing a CpG-enriched genome in the E3 gene. It was generated, propagated, and characterized using standard protocols, as previously described [51].

### Animal experiments and ethical permits

All animal experiments were reviewed and approved by the Experimental Animal Committee of the University of Helsinki and the Provincial Government of Southern Finland and the Belgian national regulations guidelines in accordance with EU Directive 2010/63/EU. The animal experiments were approved by the ethical committee for animal care of the faculty of medicine of the Université catholique de Louvain (UCL/MD/010/2019). Five weeks-old C57BL/6 mice were purchased from Envigo (Harlan, Netherlands) or by Janvier (France). Water and food were provided ad libitum.

#### Tumor implantation and tumor growth measurement

At day 0, 1 × 10^5^ B16F1 cells diluted in 100 μl of PBS were injected subcutaneously into the right flank of each mouse. Tumors were measured with an electronic digital caliper daily, starting from day 6 post tumor injection. Tumor volume was calculated as length × width × height (in mm^3^). Mice were sacrificed when the tumor volume was greater than 1500 mm^3^ or when they were in poor condition and expected to die shortly. Tumors and spleens were collected and used for further experiments. In another experiment, mice survival has been followed until the end.

#### pDNA vaccine injection and electroporation

Before each vaccine injection, mice were anesthetized with ±150 μl of a solution of 10 mg/ml ketamine (Ketalar, Pfizer, New York) and 1 mg/ml xylazine (Sigma, St. Louis, Missouri). The left paw was shaved using a rodent shaver (Aesculap Exacta shaver, AgnTho’s, Sweden). Mice were injected with 1 μg of every plasmid (pDNA), or with 1 μg of pTOP-OVA CD4-OVA CD8 irrelevant plasmid, diluted in 30 μl of PBS in the left tibialis cranial muscle. The paw was then placed between 4 mm plate caliper electrodes (BEX Co., Ltd., Japan) and electroporated (200 V/cm, 8 pulses, 20 ms with 500 ms pause between pulses). The pulses were delivered by a CUY21EX electroporator (BEX Co., Ltd., Japan). The vaccine was administered 2, 9 and 16 days after tumor injection (Fig. 1a).

#### Virus injection

Before each virus injection, mice were anesthetized in an isoflurane chamber. Then, they were injected intratumorally with 10^9^ vp CpG-rich OAd, at day 10, 12 and 14 after tumor injection. The protocol schedule of tumor, pDNA and virus injection is shown in Fig. 1a.

### Enzyme-linked ImmunoSpot (ELISPOT)

ELISpot was performed according to the manufacturer’s instruction (Immunospot, The ELISPOT source, Germany). Briefly, 3 × 10^5^ fresh splenocytes diluted in 100 μl CTL-Test medium (Immunospot, The ELISPOT source, Germany) were cultured overnight at 37 °C in anti-IFNg coated 96 well plate. For stimulation, 10 ng/μl TRP2 peptide was added to the splenocytes and incubated for 3 days. As positive control for splenocyte activation, Cell Stimulation Cocktail (Invitrogen, California) were used; PBS was used as negative control. The development of the ELISpot plate followed the manufacturer’s instruction (Immunospot, The ELISPOT source, Germany). Spots were counted by using an ELISPOT reader system (Immunospot).

### Flow cytometry analysis

NK, CD4 and CD8 T cell populations were analyzed by FACS. Tumors and spleen were surgically removed 17 days after B16F1 cell injection and FACS analysis of tumors and spleens T and NK population was performed. To prepare single cell suspensions, cells were passed through a 70 μm cell strainer (BD Falcon, New Jersey). Then, they were collected, counted using an automatic cell counter (Invitrogen, California) and washed with PBS, before adding the blocking solution with anti-CD16/CD32 antibody for 10 min on ice (clone 93, Biolegend, San Diego, California). Cells were washed and incubated for 60 min at 4 °C with the following antibodies: CD49-APC, CD335-FITC, CD11b-PerCP-Cy5.5 (for NK detection), CD3-PerCPCy5.5, CD4-PeCy7, CD8-FITC (for CD4 and CD8 detection). For staining with anti-FoxP3-PE (clone FJK-16 s, eBioscience, Thermo Fisher, Waltham, Massachusetts) or anti-IFNg-PE (clone XMG1.2, Biolegend, San Diego, California), cells were previously incubated overnight at 4 °C with a permeabilization/fixation solution (eBioscience™ Foxp3 / Transcription Factor Staining Buffer Set, Thermo Fisher, Waltham, Massachusetts). Cells were then incubated with anti-CD16/CD32 antibody for 10 min on ice (Biolegend, San Diego, California), washed and incubated for 60 min at 4 °C with anti-IFNg-PE or antiFoxP3-PE diluted in the permeabilization/fixation solution. Samples were washed with PBS fixed for 10 min with 4% formalin and, then, suspended in PBS. Sample data were acquired with FACS Fortessa or FACS Accuri (BD bioscience, Franklin Lakes, New Jersey) and analyzed with FlowJo software (FlowJo LLC, Ashland, Oregon). For the tumor analysis, the number of cells was normalized by the tumor volume (mm^3^).

### qPCR analysis

Tumors extracted at day 17 were analyzed by qPCR. Total RNA was isolated using TRIzol reagent (Thermo Fisher, Waltham, Massachusetts) and phenol separation, as previously described [14]. The quality and quantity of RNA were evaluated using a nanospectrophotometer (NanoDrop 2000, Thermo Fisher, Waltham, Massachusetts). Extracted RNA was considered pure if the 260/280 absorbance ratio of the sample was approximately 2 and the 260/230 absorbance ratio was 1.8–2.2. One microgram of RNA was reverse transcribed using a first-strand synthesis system (SuperScriptTM, Thermo Fisher, Waltham, Massachusetts) and oligo(dT) primers (Eurogentec, Liege, BE) according to the supplier’s protocol. The resulting cDNA was used as template for 40 cycles of PCR amplification. SYBR™ green real-time qPCR (GoTaq qPCR MasterMix kit, Promega, Fitchburg, Winsconsin) was conducted on a StepOne Plus Real-Time PCR System (Thermo Fisher, Waltham, Massachusetts) to detect different interleukins, chemokines, perforin and granzyme B. mRNA expression in the tumors and antigen gDNA and mRNA expression in the B16F1 cells. Analysis of the melting curves was performed to ensure purity of PCR products. The results were analyzed with StepOne Software V2.1. The mRNA expression of the cytokines was calculated relative to the corresponding expression of β-actin (reference gene) according to the delta-delta Ct method. The results were normalized compared to the mock control group. A complete list of the primers used in this study is shown in Table 2.

**Table 2:** List of the primers used in this study

Primer name		Primer sequence (5′ ➔ 3′)	Amplicon length (bp)
Granzyme B	For	GAAGCCAGGAGATGTGTGCT	183
	Rev	GCACGTTTGGTCTTTGGGTC	
Perforin	For	TCACACTGCCAGCGTAATGT	419
	Rev	AGGGCTGTAAGGACCGAGAT	
TNFa	For	CATCTTCTCAAAATTCGAGTGACAA	175
	Rev	TGGGAGTAGACAAGGTACAACCC	
IL2	For	TCACATTGACACTTGTGCTCCT	191
	Rev	CATCCTGGGGAGTTTCAGGTTC	
IL15	For	TTGGGCTGTGTCAGTGTAGG	182
	Rev	TGCAATTCCAGGAGAAAGCAGT	
IL12	For	GGAAGCACGGCAGCAGAATA	180
	Rev	AACTTGAGGGAGAAGTAGGAATGG	
IL1b	For	AACTGTTCCTGAACTCAACTGT	150
	Rev	GAGATTTGAAGCTGGATGCTCT	
IL10	For	GGTTGCCAAGCCTTATCGGA	115
	Rev	TCAGCTTCTCACCCAGGGAA	
CCL5	For	CTGCTGCTTTGCCTACCTCTC	149
	Rev	GAACCCACTTCTTCTCTGGGT	
b-actin	For	ACTCCTATGTGGGTGACGAG	206
	Rev	CATCTTTTCACGGTTGGCCTTAG	

### Additivity vs synergy analysis: the Spector’s formula

The synergy vs additivity analysis has been performed by using the Spector’s formula, as described in [24] and in Kos et al. (revised version under review). Briefly, for an additive effect, the combination index (CI) is in between +/− 2 times the standard error (SE): -2SE < CI < + 2SE; while, for a synergic effect: CI > + 2SE. The SE is defined as the derived as the square root of the total variance divided by the number of samples. The CI is defined by the following formula:

$$ CI=\ln \left(\overline{X1}\right)+\ln \left(\overline{X2}\right)-\ln \left(\overline{X1+2}\right)-\ln \left(\overline{X0}\right) $$

Where:

$$ {\displaystyle \begin{array}{l}\overline{\mathrm{X}1}=\mathrm{mean}\ \mathrm{of}\ \mathrm{the}\ \mathrm{effect}\ \mathrm{of}\ \mathrm{the}\ \mathrm{control}\ 1\ \left(\mathrm{in}\ \mathrm{our}\ \mathrm{case},\mathrm{the}\ \mathrm{mean}\ \mathrm{of}\ \mathrm{the}\ \mathrm{tumor}\ \mathrm{volumes}\ \mathrm{for}\ \mathrm{pDNA}\ \mathrm{alone}\right).\\ {}\overline{\mathrm{X}2}=\mathrm{mean}\ \mathrm{of}\ \mathrm{the}\ \mathrm{effect}\ \mathrm{of}\ \mathrm{the}\ \mathrm{control}\ 2\ \left(\mathrm{in}\ \mathrm{our}\ \mathrm{case},\mathrm{the}\ \mathrm{mean}\ \mathrm{of}\ \mathrm{the}\ \mathrm{tumor}\ \mathrm{volumes}\ \mathrm{for}\ \mathrm{OAd}\ \mathrm{alone}\right).\\ {}\overline{\mathrm{X}1+2}=\mathrm{mean}\ \mathrm{of}\ \mathrm{the}\ \mathrm{effect}\ \mathrm{of}\ \mathrm{the}\ \mathrm{combination}\ \left(\mathrm{in}\ \mathrm{our}\ \mathrm{case},\mathrm{the}\ \mathrm{mean}\ \mathrm{of}\ \mathrm{the}\ \mathrm{tumor}\ \mathrm{volumes}\ \mathrm{for}\ \mathrm{the}\ \mathrm{pDNA}+\mathrm{OAd}\ \mathrm{group}\right).\\ {}\overline{\mathrm{X}0}=\mathrm{mean}\ \mathrm{of}\ \mathrm{the}\ \mathrm{effect}\ \mathrm{of}\ \mathrm{the}\ \mathrm{non}\hbox{-} \mathrm{treated}\ \mathrm{group}\ \left(\mathrm{in}\ \mathrm{our}\ \mathrm{case}\ \mathrm{the}\ \mathrm{mean}\ \mathrm{of}\ \mathrm{the}\ \mathrm{tumor}\ \mathrm{volumes}\ \mathrm{for}\ \mathrm{the}\ \mathrm{mock}\ \mathrm{group}\right).\end{array}} $$

### Statistical analysis

Statistical analyses were performed using GraphPad Prism 7 for Windows. Survival curves were compared using a Mantel–Cox (log-rank) test. *p*-values less than 0.05 were considered statistically significant and indicated with different letters on the graphs (a, b, c, d). In particular, the presence of two different letters in two groups indicate a statistical difference (*p* < 0.05) between them; the same letter in two different groups indicates the absence of a statistical difference between these two groups. The annotation “a,b” indicates no significant differences compared to a and b statistical groups.

## Additional files

Additional file 1: pDNA vaccine design. pDNA vaccine design. Four DNA vaccines encoding one CD4 epitope (in red) and one CD8 epitope (in green). (PDF 35 kb)

Additional file 2: Expression of TRP2 and murine gp100 in B16F1 melanoma cell line. Expression of TRP2 and murine gp100 in B16F1 melanoma cell line. A) Expression of murine and human gp100 in B16F1 and B16F10 (used as a positive control). In the second line, a negative control for the primers has been performed. B) Expression of TRP2 in B16F1, B16F10 and B16F10-OVA cells (used as a positive control). In the last line, a negative control for the primers has been performed. (PDF 52 kb)

## Data Availability

The datasets used and/or analyzed during the current study are available from the corresponding author on reasonable request.

## Abbreviations

IM: Intramuscular
IT: Intratumoral
OAd: Oncolytic adenovirus
OVs: Oncolytic viruses
pDNA: Poly-epitope DNA vaccine
TAAs: Tumor associated antigens
Th: T helper
TME: Tumor microenvironment
VSV-G: vesicular stomatitis Indiana virus G protein
