# Supplementary material for: Oncolytic adenovirus drives specific immune response generated by a poly-epitope pDNA vaccine encoding melanoma neoantigens into the tumor site
Source: J Immunother Cancer. 2019 Jul 10;7:174. doi: 10.1186/s40425-019-0644-7 (PMC6621971; doi:10.1186/s40425-019-0644-7)
Supplement: Supplementary file 1 — pDNA vaccine design. pDNA vaccine design. Four DNA vaccines encoding one CD4 epitope (in red) and one CD8 epitope (in green). (PDF 35 kb) [file 40425_2019_644_MOESM1_ESM.pdf]

Oncolytic adenovirus drives specific immune response generated by a poly-epitope pDNA vaccine encoding melanoma neoantigens into the tumor site

Alessandra Lopes[1], Sara Feola[2], Sophie Ligot[1], Manlio Fusciello[2], Gaëlle Vandermeulen[1], Véronique Préat[1#], Vincenzo Cerullo[2#]

Supplementary data 1

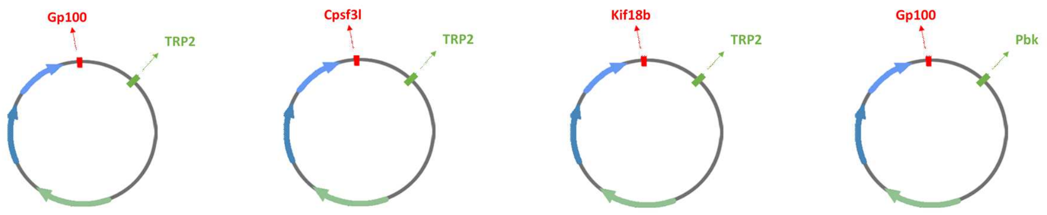

Supplementary data 1: pDNA vaccine design. Four DNA vaccines encoding one CD4 epitope (in red) and one CD8 epitope (in green).
