# Supplementary material for: Oncolytic adenovirus drives specific immune response generated by a poly-epitope pDNA vaccine encoding melanoma neoantigens into the tumor site
Source: J Immunother Cancer. 2019 Jul 10;7:174. doi: 10.1186/s40425-019-0644-7 (PMC6621971; doi:10.1186/s40425-019-0644-7)
Supplement: Supplementary file 2 — Expression of TRP2 and murine gp100 in B16F1 melanoma cell line. Expression of TRP2 and murine gp100 in B16F1 melanoma cell line. A) Expression of murine and human gp100 in B16F1 and B16F10 (used as a positive control). In the second line, a negative control for the primers has been performed. B) Expression of TRP2 in B16F1, B16F10 and B16F10-OVA cells (used as a positive control). In the last line, a negative control for the primers has been performed. (PDF 52 kb) [file 40425_2019_644_MOESM2_ESM.pdf]

Oncolytic adenovirus drives specific immune response generated by a poly-epitope pDNA vaccine encoding melanoma neoantigens into the tumor site

Alessandra Lopes[1], Sara Feola[2], Sophie Ligot[1], Manlio Fusciello[2], Gaëlle Vandermeulen[1], Véronique Préat[1#], Vincenzo Cerullo[2#]

Supplementary data 2

A)

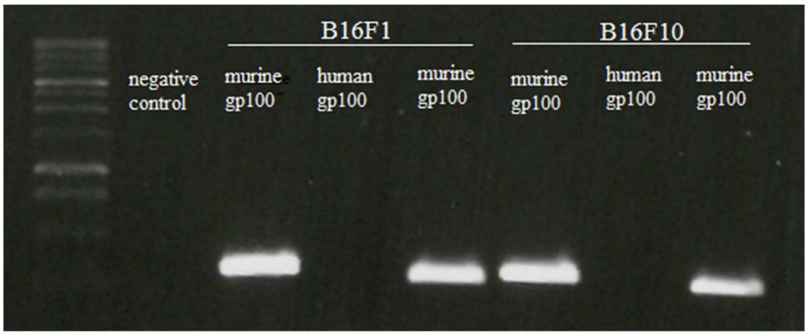

B)

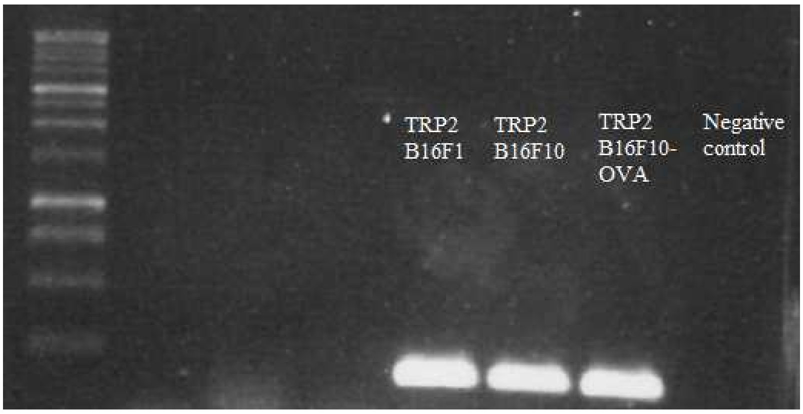

Supplementary data 2: Expression of TRP2 and murine gp100 in B16F1 melanoma cell line.

A) Expression of murine and human gp100 in B16F1 and B16F10 (used as a positive control). In the second line, a negative control for the primers has been performed. Ladder of 1 Kb.

B) Expression of TRP2 in B16F1, B16F10 and B16F10-OVA cells (used as a positive control). In the last line, a negative control for the primers has been performed. Ladder of 1Kb.
